# Assessment of clinical feasibility:offline adaptive radiotherapy for lung cancer utilizing kV iCBCT and UNet++ based deep learning model

**DOI:** 10.1002/acm2.14582

**Published:** 2024-11-29

**Authors:** Hongwei Zeng, Qi Chen, Xiangyu E, Yue Feng, Minghe Lv, Su Zeng, Wenhao Shen, Wenhui Guan, Yang Zhang, Ruping Zhao, Shaobin Wang, Jingping Yu

**Affiliations:** ^1^ Department of Radiotherapy Shuguang Hospital Affiliated to Shanghai University of Chinese Traditional Medicine Shanghai China; ^2^ MedMind Technology Co Ltd Beijing China; ^3^ Department of Biomedical Engineering School of Medicine Tsinghua University Beijing China

**Keywords:** dose verification, kV iCBCT, lung cancer, offline adaptive radiotherapy, UNet++ model

## Abstract

**Background:**

Lung cancer poses a significant global health challenge. Adaptive radiotherapy (ART) addresses uncertainties due to lung tumor dynamics. We aimed to investigate a comprehensively and systematically validated offline ART regimen with high clinical feasibility for lung cancer.

**Methods:**

This study enrolled 102 lung cancer patients, who underwent kV iterative cone‐beam computed tomography (iCBCT). Data collection included iCBCT and planning CT (pCT) scans. Among these, data from 70 patients were employed for training the UNet++ based deep learning model, while 15 patients were allocated for testing the model. The model transformed iCBCT into adaptive CT (aCT). Clinical radiotherapy feasibility was verified in 17 patients. The dosimetric evaluation encompassed GTV, organs at risk (OARs), and monitor units (MU), while delivery accuracy was validated using ArcCHECK and thermoluminescent dosimeter (TLD) detectors.

**Results:**

The UNet++ based deep learning model substantially improved image quality, reducing mean absolute error (MAE) by 70.05%, increasing peak signal‐to‐noise ratio (PSNR) by 17.97%, structural similarity (SSIM) by 7.41%, and the Hounsfield Units (HU) of aCT approaching a closer proximity to pCT compared to kV iCBCT. There were no significant differences observed in the dosimetric parameters of GTV and OARs between the aCT and pCT plans, confirming the accuracy of the dose maps in ART plans. Similarly, MU manifested no notable disparities, underscoring the consistency in treatment efficiency. Gamma passing rates for intensity‐modulated radiation therapy (IMRT) and volumetric‐modulated arc therapy (VMAT) plans derived from aCT and pCT exceeded 98%, while the deviations in TLD measurements (within 2% to 7%) also exhibited no significant differences, thus corroborating the precision of dose delivery.

**Conclusion:**

An offline ART regimen utilizing kV iCBCT and UNet++ based deep learning model is clinically feasible for lung cancer treatment. This approach provides enhanced image quality, comparable treatment plans to pCT, and precise dose delivery.

## 1 INTRODUCTION

Lung cancer remains a significant global health burden, with radiotherapy playing a crucial role in its treatment paradigm.^1^, ^2^ Despite advancements in radiotherapy techniques, the persistent challenge of uncertainty in radiotherapy, particularly due to the dynamic nature of lung tumor anatomy, continues to limit treatment efficacy.^3^ Adaptive radiotherapy (ART) has emerged as a promising strategy to address these challenges by dynamically adjusting treatment plans based on patient‐specific anatomical variations over the course of treatment.^4^

Online ART employs real‐time feedback from cone‐beam computed tomography (CBCT) scans to dynamically adjust treatment protocols, effectively managing tumor mobility challenges.^5^ Its efficacy is contingent upon sophisticated image‐guidance systems and complex treatment planning software, which increase technical demands and costs, potentially prolonging radiation sessions and affecting patient tolerance and adherence. In contrast, offline ART facilitates systematic assessment of anatomical changes and comprehensive adaptation through periodic updates of treatment plans using scheduled imaging data.^6^ Particularly pertinent in the context of lung cancer, characterized by significant respiratory motion and anatomical variability, offline ART stands out as a favorable option due to its feasibility and practicality in addressing these challenges.^7^ This approach harbors the potential to enhance treatment outcomes by accommodating variations in tumor size, shape, and position, while minimizing unnecessary irradiation of healthy tissues.

Central to the success of offline ART lies the synergy between advanced imaging modalities such as CBCT and state‐of‐the‐art deep learning models like UNet++, which are capable of transforming CBCT images into adaptive‐CT images (aCT).^8^ CBCT offers high‐resolution volumetric imaging, indispensable for monitoring anatomical changes throughout treatment, and is further enhanced by deep learning algorithms capable of generating aCT. The UNet++ based deep learning model, an evolution of the UNet architecture, excels in medical image segmentation. With its intricate design, including skip connections and densely connected convolutional blocks, UNet++ accurately delineates anatomical structures from complex and noisy CBCT images, enabling high‐quality aCT generation.^9^ Its superiority lies in handling intricacies within medical images, effectively addressing challenges like anatomical variability and image artifacts crucial for accurate aCT production. By leveraging contextual information through skip connections and dense blocks, UNet++ produces precise segmentations critical for offline ART, especially in aCT generation.^10^ Furthermore, its adaptability to varying imaging conditions and robust performance across diverse datasets make it suitable for integration into offline ART workflows, particularly where accurate aCT is essential.^11^, ^12^

This study aimed to evaluate the clinical feasibility of offline ART for lung cancer, utilizing kV iterative CBCT (iCBCT) and UNet++‐based deep learning model. We performed a comprehensive assessment with clinical datasets and rigorous validation methods, focusing on various aspects such as the image quality of the reconstructed aCT, the accuracy of dose distribution in adaptive plans designed by the treatment planning system (TPS), and the precision of actual dose delivery.

## 2 METHODS AND MATERIALS

### 2.1 Data collection

The study enrolled a total of 102 patients diagnosed with primary lung cancer, all of whom received radiotherapy during 2022–2023. The kV iCBCT scans for each patient, along with their corresponding planning CT scans (pCT), were collected for analysis. The pCT data were acquired using Siemens SOMATOM Confidence 20 large‐aperture CT scanner, with a tube voltage of 120 kV and a slice thickness of 5 mm. The kV iCBCT data were acquired utilizing Varian Halcyon 3.0 linear accelerator, following a Thorax scanning protocol. The iCBCT scans were performed at a tube voltage of 125 kV, a tube current of 294 mAs, and a reconstruction resolution of 512 × 512 pixels. The reconstruction thickness was set to 2 mm, and the image quality enhancement function was activated during acquisition.^13^

### 2.2 UNet++‐based deep learning model development

**FIGURE 1:**
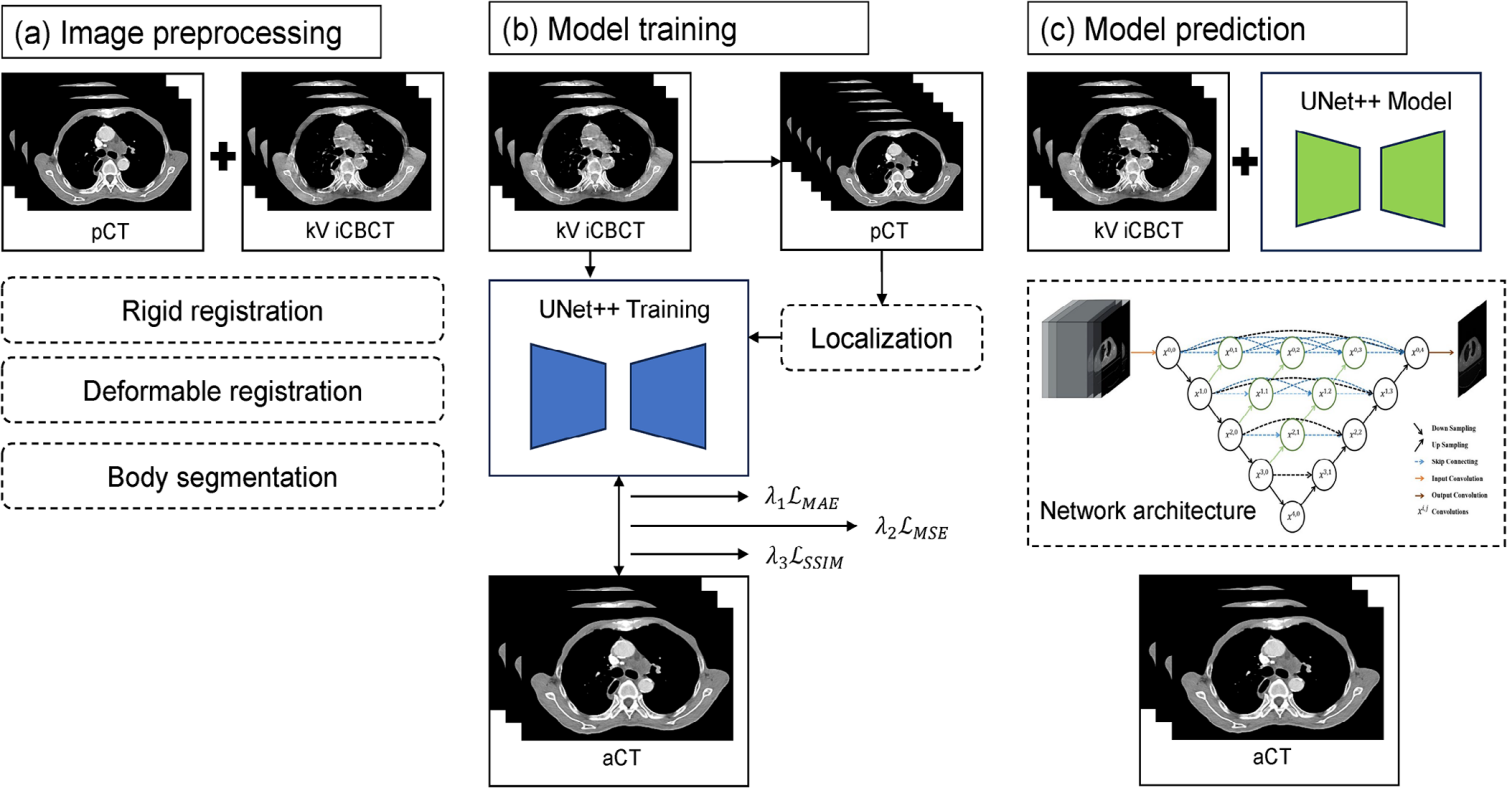
Framework of UNet++‐based deep learning model employed in this study.

This study leveraged data from 70 lung cancer patients, encompassing pCT and iCBCT data sourced from the inaugural radiotherapy session for each participant. In the data preprocessing stage, as shown in Figure 1a, registration fusion was conducted between iCBCT and pCT. Rigid registration was employed to globally adjust the position, while elastic registration was used to align corresponding organs. iCBCT was associated with localized irradiation, while pCT entailed global irradiation. Consequently, delineation of the body on iCBCT was performed to outline the region of interest. Subsequently, the intensity range of CT images was adjusted to [−1000 Hounsfield Units (HU) to 3000 HU], and the data underwent Z‐Score normalization.

UNet++ epitomizes an encoder‐decoder paradigm, replete with dense skip connections, poised to fortify the fusion of profound and superficial features, thereby amplifying the learning aptitude and generalization acumen of features. In the decoder module of UNet++, feature maps are adjoined with their corresponding counterparts from the encoder's levels, predicated upon the fusion outcomes of divergent feature levels, thereby birthing skip connections. As depicted in Figure 1b,c), the synthesized model's encoder in this inquiry, harnessed ResNet50 as the backbone network, wherein 5 $x^{i,0}$ delineated five distinct scale features extracted by the backbone network, accentuating the crux on feature extraction and assimilation. Subsequently, the decoder module served the vital purpose of reinstating image features and generating predictive images. This experiment adopted a composite loss function (L), embracing three pivotal components: mean absolute error (MAE), mean square error (MSE), and structural similarity (SSIM) loss. The weighting coefficients of these losses were finetuned via hyperparameters $\lambda_{1}$, $\lambda_{2}$, and $\lambda_{3}$ to ensure the image quality of the final output. Following iterative experimentation and cross‐validation, these coefficients were calibrated to $\lambda_{1}$ = 100, $\lambda_{2}$ = 1, and $\lambda_{3}$ = 1. Consequently, our ultimate loss function, denoted as Formula (1), solely appraised the losses within the body region for MAE and MSE. Owing to the salient dissimilarities in machinery and imaging principles between iCBCT and CT, SSIM was enlisted to optimize and harmonize the brightness, contrast, and structure of the output results.

(1) $$L=\lambda_{1}L_{\mathrm{MAE}}+\lambda_{2}L_{\mathrm{MSE}}+\lambda_{3}L_{\mathrm{SSIM}}$$

All training endeavors were actualized leveraging PyTorch 1.10.1 (Facebook, California, USA) and executed on the RTX A6000 GPU (NVIDIA, California, USA), endowed with 48GB of memory. The model's input data dimensions stood at 3 × 512 × 512, while the resultant output dimensions were 1 × 512 × 512. During the training phase, stochastic data augmentation was invoked, featuring a probability of 0.5, inclusive of rotational transformations, translations, and horizontal flips. Training was steered using the Adam optimizer, initializing with a learning rate of $1e^{-4}$, and persisted for a cumulative span of 200 epochs.

### 2.3 Image quality assessment

The iCBCT, aCT, and pCT images from 17 lung cancer patients were utilized for model performance evaluation. MAE, SSIM, and peak signal‐to‐noise ratio (PSNR), commonly employed as evaluation metrics for synthetic CT, were used to assess the quality of the generated composite images, as indicated by formulas (2), (3), and (4) respectively.^14^ A smaller MAE indicated a diminished disparity in HU values between the two images. SSIM possessed a maximum value of 1, whereby higher values signified enhanced image quality and augmented similarity between the two images. A higher PSNR value corresponded to reduced distortion and superior image quality, thereby indicating increased similarity between the two images.

(2) $$\mathrm{MAE}\left(I_{1},I_{2}\right)=\sum_{x,y,z}^{n_{i}n_{j}n_{k}}\left|I_{1}\left(x,y,z\right)-I_{2}\left(x,y,z\right)\right|$$

(3) $$\mathrm{SSIM}\left(I_{1},I_{2}\right)=\frac{\left(2\mu_{I_{1}}\mu_{I_{2}}+c_{1}\right)\left(2\sigma_{I_{1},I_{2}}+c_{2}\right)}{\left(\mu_{I_{1}}^{2}+\mu_{I_{2}}^{2}+c_{1}\right)\left(\sigma_{I_{1}}^{2}+\sigma_{I_{2}}^{2}+c_{2}\right)}$$

(4) $$\mathrm{PSNR}\left(I_{1},I_{2}\right)=10\times \log_{10}\left(\frac{MAX^{2}}{\sum_{x,y,z}^{n_{i}n_{j}n_{k}}\frac{{\left|I_{1}\left(x,y,z\right)-I_{2}\left(x,y,z\right)\right|}^{2}}{n_{i}n_{j}n_{k}}}\right)$$

In this context, $I_{1}$ and $I_{2}$ represented the two images, I(x,y,z) denoted the HU value of a point in the image, and $n_{i}n_{j}n_{k}$ represented the total number of pixels in the image. *MAX* denoted the maximum HU value in the image. μ and σ represented the mean and standard deviation of HU values in the image.

### 2.4 Plans dosimetric evaluation

This study involved 17 lung cancer patients, selecting both the iCBCT and pCT images acquired within a 24‐h interval from each patient. This selection aimed to align with the clinical context of offline ART. Implementing the UNet++‐based deep learning model, the iCBCT images were transformed into aCT. The gross tumor volume (GTV) in both aCT and pCT was delineated by a radiation oncologist, while automated delineation software was employed to delineate critical organs, including the bilateral lungs, heart, and spinal cord. Treatment plans were designed using the Eclipse v15.6 TPS (Varian, California, USA), prescribing 60 Gy/30fx. Consistency was maintained in various parameters such as field parameters, optimization algorithms, dose grid, optimization criteria, and iteration numbers for both the aCT and pCT plans. To facilitate precise dosimetric analysis, aCT was rigidly registered to pCT, enabling the transfer of dose distributions from aCT to pCT. The assessed dosimetric parameters encompassed the mean dose (*D*_mean_) of GTV, homogeneity index (HI), conformity index (CI), and gradient index (GI), as indicated by formulas (5), (6), and (7). In this context, V_GTV,ref_ indicated the volume covered by the prescription dose, where V_GTV_ was the target volume, and V_ref_ was the total volume covered by the prescription dose. *D*_2%_, *D*_98%_, and *D*_50%_ represented doses received by 2%, 98%, and 50% of the target volume, respectively. V_50%_ received half of the prescription dose, while V_100%_ received the full prescription dose.

(5) $$\mathrm{CI}=\frac{V_{\mathrm{GTV},\mathrm{ref}}\times V_{\mathrm{GTV},\mathrm{ref}}}{V_{\mathrm{GTV}}\times V_{\mathrm{ref}}}$$

(6) $$\mathrm{HI}=\frac{D_{2\%}-D_{98\%}}{D_{50\%}}$$

(7) $$\mathrm{GI}=\frac{V_{50\%}}{V_{100\%}}$$

Parameters evaluating organs at risk (OARs) included the mean dose to bilateral lungs and ipsilateral lung, volumes receiving doses of 5  Gy (V_5_), 20  Gy (V_20_), and 30  Gy (V_30_), *D*_mean_ of heart, and maximum dose (*D*_max_) of spinal cord. Additionally, total monitor units (MU) also required evaluation to analyze differences in treatment efficiency.

### 2.5 Delivery accuracy verification

Plan evaluation is a predictive software‐based analytical approach, while verifying the accuracy of delivered radiotherapy dose is an indispensable step in the clinical application of any new radiotherapy technology. Initially, the study utilized the ArcCHECK (SunNuclear, Florida, USA) three‐dimensional dose verification system to validate the gamma passing rate of plans developed based on aCT, encompassing 85 intensity‐modulated radiation therapy (IMRT) plans and 85 volumetric‐modulated arc therapy (VMAT) plans.^15^ Within the IMRT and VMAT plans, there were 17 plans each with dose grids of 1, 2, 2.5, 3, and 4 mm. The ArcCHECK three‐dimensional dose verification system, which included the SNC v6.2.3 analysis software (SunNuclear, Florida, USA), consists of a cylindrical water‐equivalent phantom and 1386 semiconductor detectors spaced at 1 cm intervals, with an effective detection area of 0.8 × 0.8 mm^2^. Following background calibration of the ArcCHECK phantom, adjustments were made for symmetry along the X and Y axes, and dose correction was performed at the center of a 10 × 10 cm^2^ field.^16^ The gamma analysis successfully meets criteria with a dose threshold of 10%, a dose tolerance (DT)/distance to agreement (DTA) set at 3%/3 mm, yielding a plan verification pass rate exceeding 97%.

Additionally, thermoluminescent dosimeter (TLD) has been extensively employed in numerous studies related to radiotherapy dose measurement.^17^, ^18^ In this study, a heterogeneous anthropomorphic phantom (702 D, CIRS, Norfolk), combined with TLD detectors, was used to capture actual irradiation dose across various density tissues within the chest, including lung, heart, thoracic vertebrae, rib, and breast. The model comprises 38 slices and two breasts, with each slice 25 mm thick and containing multiple 5 mm diameter holes.^19^ TLD detectors used in the study underwent calibration under simulated irradiation conditions at the National Institute of Metrology in China. Calibration parameters involved 6 MVFFF photons with doses of 0.1 , 1 , and 2 Gy. Dose calibration experiments revealed notable dose linearity of TLD detectors across different doses under identical radiation and energy settings, thus establishing the reliability of calibration coefficients for dose measurements in linear accelerators. This approach rigorously assessed the accuracy of planned dose delivery based on aCT in real world scenarios.

### 2.6 Statistics analysis

The statistical analysis was conducted utilizing SPSS 21 software (IBM, NYC, USA), wherein data were represented as mean ± standard deviation ($\bar{x}\pm s$). Shapiro‐Wilk (S‐W) tests were performed individually for each dataset, with a *p *> 0.05 indicating adherence to the assumption of normal distribution. Paired sample *t*‐tests were conducted when the data met the normality assumption. Conversely, non‐parametric tests were applied to datasets that did not satisfy the normality assumption. Statistical significance was established at *p *< 0.05.

## 3 RESULTS

**FIGURE 2:**
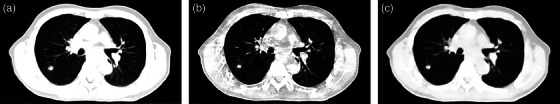
Qualitative results of images generated by different methods. (a) was the pCT derived from localization CT scans. (b) was the original iCBCT. (c) was the aCT produced by the UNet++ based deep learning model.

**FIGURE 3:**
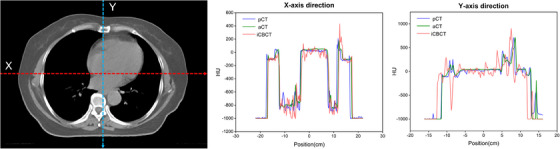
HU dotted line profile map and histograms of HU values for test patient case. The left image depicted cross‐sectional views intersected by two dotted lines, with red representing the X‐axis and blue representing the Y‐axis. In the middle and right panels, the HU value distributions along the X‐axis and Y‐axis was respectively presented for pCT, aCT, and iCBCT.

Figure 2 presents a comparative analysis of the aCT, original iCBCT, and pCT images derived from the UNet++‐based deep learning model employed in this study. Notably, discernible improvements in overall image quality were observed, particularly in the suppression of artifacts within the iCBCT images, thereby resulting in a closer resemblance to the pCT scans. A quantitative assessment of image quality between aCT and iCBCT across the entire test dataset is provided in Table 1. The aCT images exhibited a significant decrease in MAE from 96.20 ± 11.61 to 53.56 ± 9.74 HU (*p *< 0.001), accompanied by an increase in PSNR from 29.40 ± 1.58  to 33.13 ± 1.55 dB (*p *< 0.001), and SSIM from 0.81 ± 0.04 to 0.86 ± 0.04 (*p *< 0.001) compared to iCBCT. These alterations in MAE, PSNR, and SSIM, representing increases of 70.05%, 17.97%, and 7.41%, respectively, indicated significant enhancements across all evaluated metrics. Additionally, Figure 3 demonstrates that the distribution trend of HU passing through the dotted lines (X and Y axis) in the synthesized aCT aligned more closely with pCT, thereby emphasizing the capability of the UNet++‐based deep learning model to significantly improve the accuracy of synthesized image HU values.

**TABLE 1:** Comparison of MAE, PSNR, and SSIM between the original iCBCT and aCT generated by UNet++‐based deep learning model, using pCT as the reference standard.

	iCBCT	aCT		
Items	Value	Value	Amelioration	*p*
MAE(HU)	96.20 ± 11.61	53.56 ± 9.74	70.05%	<0.001^*^
PSNR(dB)	29.40 ± 1.58	33.13 ± 1.55	17.97%	<0.001^*^
SSIM	0.81 ± 0.04	0.86 ± 0.04	7.41%	<0.001^*^

* Denoted that the difference was statistically significant.

Table 2 systematically illustrates the variations in dose between the two types of dose maps for GTV and OARs. These two dose distributions were derived from pCT and aCT generated by the UNet++‐based deep learning model, respectively. Following the establishment of uniform optimization parameters, the dose distribution from aCT was overlaid onto pCT for comparative evaluation. The variances in *D*_mean_, HI, and CI within the GTV were not statistically significant. However, the GI value decreased from 6.48 ± 1.76 in the pCT‐dose map to 5.92 ± 1.51 in the aCT‐dose map (*p *< 0.05). Among OARs, no significant differences were observed in all dose parameters. Moreover, there were no statistically significant disparities in MU between the two planning methodologies.

**TABLE 2:** Dosimetry results of GTV and OARs in the diverse dose maps.

Items	Dosimetry parameters	pCT‐dose map	aCT‐dose map	*p*
GTV	*D*_mean_(Gy)	61.28 ± 0.38	61.48 ± 0.65	0.136
	GI	6.48 ± 1.76	5.92 ± 1.51	0.027^*^
	HI	0.05 ± 0.02	0.06 ± 0.02	0.247
	CI	1.26 ± 0.28	1.22 ± 0.16	0.943
Bilateral lungs	*D*_mean_(Gy)	7.38 ± 2.88	7.37 ± 2.99	0.463
	V_5_(%)	37.96 ± 17.06	37.99 ± 17.14	0.889
	V_20_(%)	10.39 ± 5.50	10.30 ± 5.64	0.250
	V_30_(%)	4.90 ± 2.71	4.84 ± 2.78	0.121
Ipsilateral lung	*D*_mean_(Gy)	11.69 ± 4.35	11.63 ± 4.42	0.394
	V_5_(%)	49.34 ± 16.52	49.29 ± 16.91	0.831
	V_20_(%)	21.06 ± 10.53	21.11 ± 10.68	0.745
	V_30_(%)	11.33 ± 6.78	11.25 ± 6.78	0.778
Heart	*D*_mean_(Gy)	2.74 ± 2.88	2.59 ± 2.89	0.981
Spinal cord	*D*_max_(Gy)	24.08 ± 12.05	24.12 ± 12.02	0.492
MU		738.51 ± 324.22	741.66 ± 320.60	0.730

* Denoted that the difference was statistically significant.

The pCT‐dose map illustrated the plans dose distribution derived from the positioning CT scan. In contrast, the aCT‐dose map depicted the plans dose distribution based on the synthetic CT, synthesized using the UNet++‐based deep learning model. After undergoing rigid registration with the positioning CT scan, the dose distribution was overlaid onto the positioning CT framework.

**FIGURE 4:**
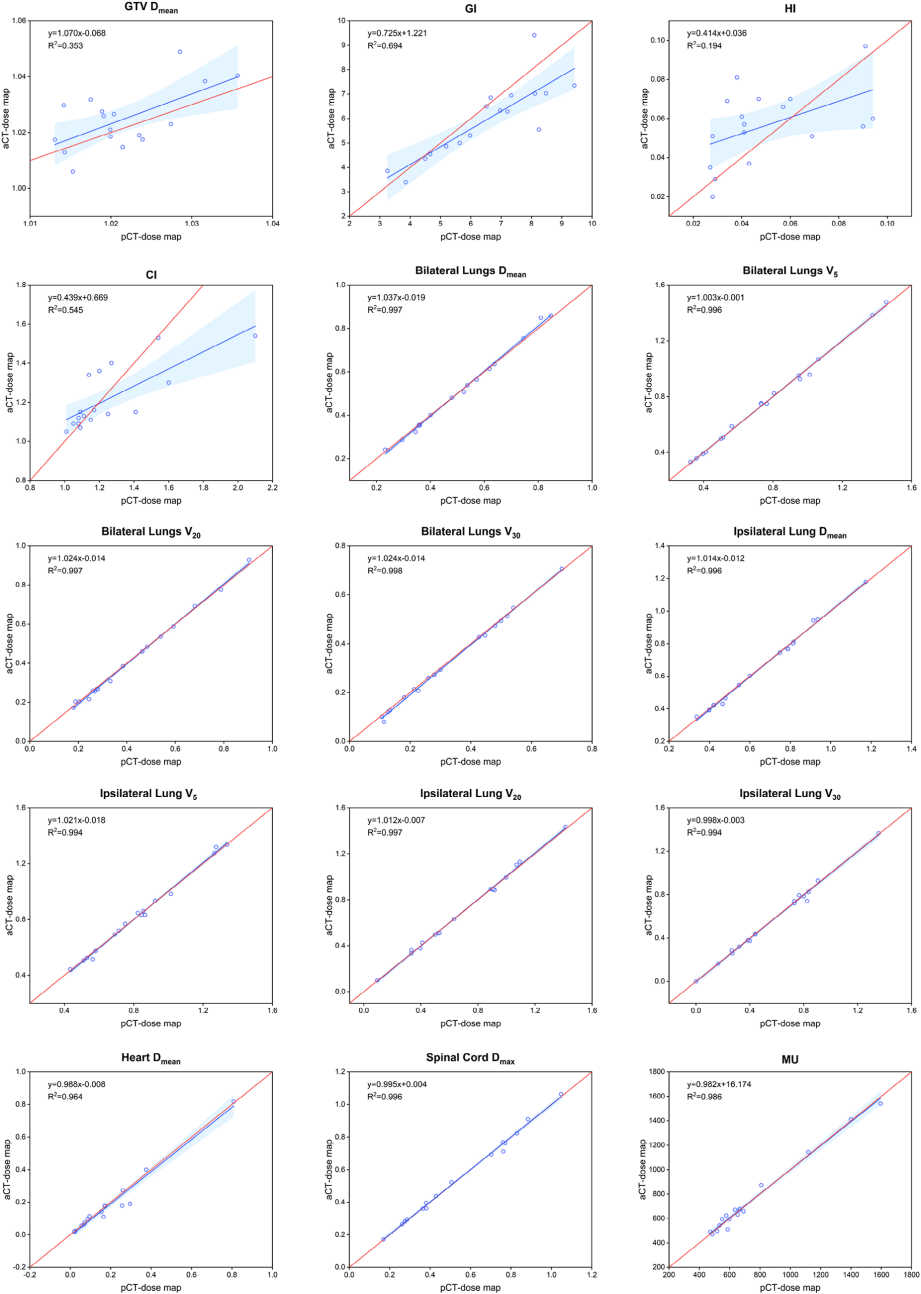
The dose‐volume distribution scatter plot and linear regression curves of dose‐volume parameters for 17 lung cancer patients. The formula for the red curve was y = x. The blue range represented the 95% confidence interval of the linear regression curve.

The dose‐volume parameters of GTV and OARs in both the pCT‐dose map and aCT‐dose map were normalized, utilizing division by their respective prescribed doses. This normalization aimed to ascertain the relative dose distribution of GTV and OARs in the two different dose maps. Simultaneously, linear regression analysis was employed to the dose parameters using the least squares method. As illustrated in Figure 4, the linear regression curves for GTV, encompassing *D*_mean_, GI, HI, and CI, exhibited relatively poor alignment with the identity line (y = x). Conversely, the linear regression curves for all OARs dose‐volume parameters, as well as the plan MU, demonstrated a high degree of concordance with the identity line, with all *R* ^2^ values exceeding 0.99.

Table 3 presents the gamma passing rate parameters of the IMRT and VMAT plans, which were formulated based on aCT, with varying dose grid sizes on the ArcCHECK platform. Across dose maps, all IMRT plans consistently achieved an average gamma passing rate exceeding 99.1%, regardless of the dose grid size employed, encompassing the clinically conventional 2.5  mm grid and other variations. The average gamma passing rate for the VMAT plans had declined to a minimum of 98.2% when using a 4  mm dose grid size.

**TABLE 3:** The gamma passing rate of IMRT plans and VMAT plans with various dose grids on the ArcCHECK platform.

Dose grid	Gamma passing rate of IMRT plans (%)	Gamma passing rate of VMAT plans (%)
1 mm	99.9 ± 0.1	99.8 ± 0.1
2 mm	99.6 ± 0.3	99.3 ± 0.5
2.5 mm	99.6 ± 0.6	99.2 ± 0.6
3 mm	99.3 ± 0.4	99.0 ± 0.7
4 mm	99.2 ± 0.4	98.2 ± 0.5

**FIGURE 5:**
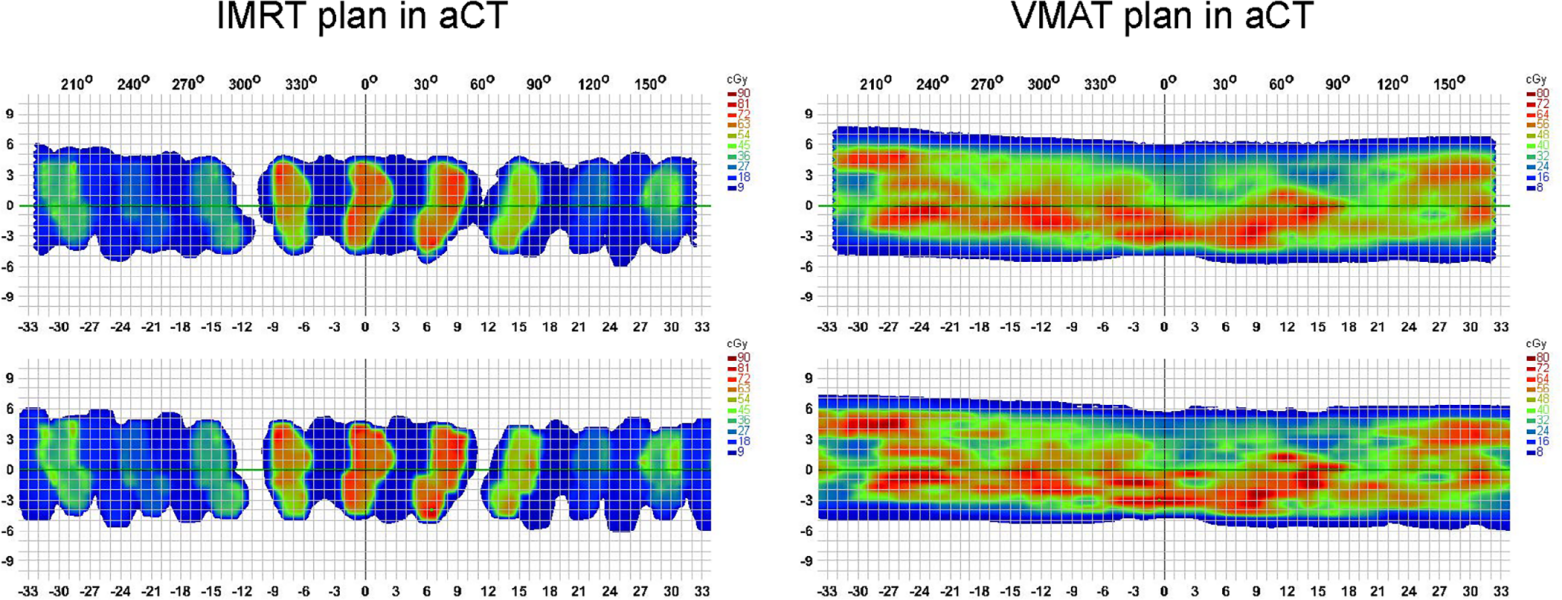
ArcCHECK measured does field compared with TPS calculated dose field of IMRT and VMAT plans in aCT. The upper section displayed the tangible dose measurement obtained through ArcCHECK for both IMRT and VMAT plans. The lower section displayed the dose calculation executed by TPS for both IMRT and VMAT plans.

Figure 5 depicts a single irradiation dose distribution analysis of the representative IMRT plan and VMAT plan. It was evident that, in both the IMRT and VMAT plans, the dose distribution obtained by ArcCHECK closely corresponded to the planned dose distribution by TPS, whether for overall cold/hot spot dose distribution or dose distribution at the boundaries.

**FIGURE 6:**
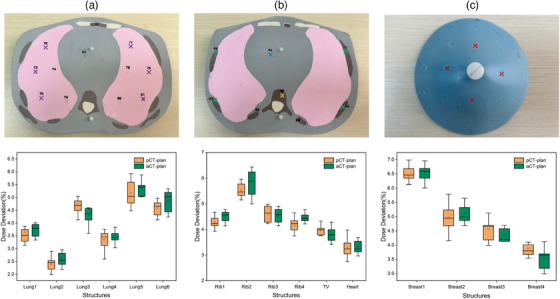
The exact measurement points of TLD for several slices, along with the dose deviations of organs between pCT‐plan and aCT‐plan. The six purple markers in (a) represented bilateral lungs. The four green markers, one yellow marker and one blue marker in (b) represented ribs, thoracic vertebrae (TV), and heart respectively. The four red markers in (c) represented breast. The pCT‐plan referred to the plan based on positioning CT, showing deviation between actual TLD‐collected dose and TPS‐calculated dose. The aCT‐plan referred to the plan based on aCT synthesized using the UNet++‐based deep learning model, showing deviation between actual TLD‐collected dose and TPS‐calculated dose.

In the validation experiments that assessed dose delivery accuracy for the ACT plan using TLD detectors and heterogeneous anthropomorphic phantom, the discrepancy between actual measured dose and TPS‐calculated dose at various organ measurement points is shown in Figure 6. Comparative analysis with the pCT plan revealed that the dose errors associated with the aCT plan demonstrated consistent behavior across all measurement points. Importantly, statistical analysis indicated no significant deviation between the two plan types (*p *> 0.05). Given the administration of a single 2 Gy dose, the radiation dosage recorded at all measurement points was notably reduced. Furthermore, the deviation between the actual measured dose and the TPS‐calculated dose for the aCT plan fell within the range of 2%–7%, mirroring the findings observed for the pCT plan.

## 4 DISCUSSION

Previous research has investigated various methodologies related to online ART. For instance, Sibolt et al. conducted a retrospective analysis of online ART for lung cancer patients using daily CBCT.^20^ While their findings suggested improved treatment precision, they highlighted challenges associated with extended treatment durations and increased resource requirements. These observations were consistent with some other studies,^21^, ^22^ which emphasized the obstacles and limitations encountered in online ART, especially in managing technical requirements and patient adherence. In contrast, this study firstly emphasized the clinical feasibility and potential of offline ART for lung cancer using kV iCBCT combined with the UNet++‐based deep learning model. Through a thorough evaluation of our methodology, including data acquisition, construction of the deep learning model, assessment of image quality, evaluation of plan dosage metrics, and validation of radiotherapy accuracy, we confirmed that the reconstructed aCT, enhanced by UNet++ algorithm, represented a viable strategy for offline ART for lung cancer. In contrast to online ART, which relies on real‐time imaging feedback from linear accelerator and subsequent dose distribution re‐optimization, our offline approach allowed for periodic adjustments based on predetermined imaging data, thereby reducing hardware technical requirements, improving treatment efficacy, and minimizing irradiation to healthy tissues.

The integration of advanced imaging modalities with the UNet++‐based deep learning algorithms represented a pivotal innovation in this study. The kV iCBCT reconstruction protocol on the Halcyon 3.0 linear accelerator platform (Varian, California, USA) incorporated both Acuros CTS and statistical reconstruction algorithms, significantly improving soft tissue resolution in the chest CBCT images.^23^, ^24^ Meanwhile, there has been rapid development in deep learning based methods in recent years, yielding promising results. A notable observation is the frequent utilization of the Cycle‐GAN‐based models for pseudo‐CT synthesis tasks. Although Cycle‐GAN enables the training of models with unpaired image data, it is essential to note that it may occasionally generate structures in pseudo‐CT images that do not correspond to actual features present in the original CBCT images.^25^, ^26^ The UNet++‐based deep learning model in this study differed from conventional two‐dimensional models by using multi‐channel images consisting of the slice to be synthesized and its two adjacent slices as inputs. These adjacent slices provided contextual information to the network, allowing it to effectively capture and represent features in medical images, thereby significantly enhancing the model's efficacy and fitting capability. Proficient at capturing intricate details and features in the CBCT scans, the UNet++‐based model efficiently learned and portrayed complex patterns in the data, thereby reducing reconstruction errors and suppressing noise and artifacts inherent in original CBCT scans.^27^ Moreover, the model improved the structural similarity between the generated the aCT images and the reference pCT images by accurately delineating anatomical structures and preserving spatial relationships, ensuring the preservation of crucial structural details, thereby enhancing the clinical utility and interpretability of the images.

This study not only explored advanced image processing but also comprehensively covered the entire workflow of offline ART for lung cancer. The consistency of dosimetric parameters and treatment efficiency between the aCT‐plans developed using the UNet++‐based deep learning model and the conventional pCT‐plans confirmed the accuracy of offline ART treatment planning. Despite potential anatomical variations among patients, adaptive plans generated using aCT remained clinically equivalent to traditional plans based on the localization CT scans, thus demonstrating the robustness of offline ART methodologies. This provided essential assurance for the clinical application of ART for lung cancer patients, ensuring not only effective management of anatomical changes but also the ability to update treatment plans based on the CBCT data throughout the entire treatment process, thereby reducing toxicity to surrounding healthy tissues.^28^

The gamma passing rates for IMRT and VMAT plans based on aCT were over 98%. Elevated gamma passing rates served as a quality assurance metric, validating the reliability and robustness of treatment plan conversion from the planning phase to delivery. TLD detector measurements constituted pivotal verification steps to ensure treatment precision and consistency. Although TLD detectors are not typically employed as routine radiation dose measurement tools in clinical quality control due to the rigorous annealing and response measurement protocols required, they offer measurement accuracy within the range of 2%–3%.^29^ These detectors independently verified whether the administered dose aligned with the planned dose, thereby validating the integrity of the treatment process and ensuring patient safety. In this study, the dose deviation between aCT plan and pCT plan was found to be comparable, ranging from 2% to 7%, with the majority of point errors within 5%. These deviations can be attributed to factors such as positioning reproducibility, multi‐leaf collimator (MLC) resolution, and the relatively small volume of measurement points. Despite these variations, the pronounced consistency between the actual delivered dose based on the aCT plan and TPS dose provided compelling evidence substantiating the clinical application of offline ART as a lung cancer treatment modality, indicating that treatment plans crafted using aCT could effectively guide radiation therapy implementation, thereby ensuring the desired dose distribution throughout the treatment continuum. Verification of dose delivery accuracy underscored the integrity and safety of the offline adaptive scheme proposed in this study for optimizing lung cancer patient treatment outcomes.

There were some limitations to this study. First, 102 patients were enrolled, and a larger and more diverse sample would enhance the generalizability of the findings. Second, the research in a single clinical center lacked external validation. Thirdly, this was a retrospective study, which might not capture long‐term outcomes and complications associated with offline ART.

Moving forward, further optimizing deep learning algorithms, enhancing imaging protocols, and integrating multimodal imaging data constitute promising avenues for exploration. Additionally, exploring the long‐term efficacy and cost‐effectiveness of offline ART in prospective clinical trials is essential for guiding clinical practice.

## 5 CONCLUSION

This study proposed a feasible offline ART regimen for lung cancer, utilizing kV iCBCT and the UNet++‐based deep learning model. We demonstrated improved image quality, comparable treatment plans to pCT, and accurate dose delivery. This approach holds promise in optimizing outcomes while minimizing toxicity. Prospective trials and algorithm refinement are needed for wider adoption.

## AUTHOR CONTRIBUTIONS

*Conception and design*: Jingping Yu, Shaobin Wang, and Hongwei Zeng. *Collection and assembly of data*: Wenhao Shen, Wenhui Guan, and Yang Zhang. *Model construction and optimization*: Hongwei Zeng and Qi Chen. *Data analysis and interpretation*: Xiangyu E, Yue Feng, Minghe Lv, and Su Zeng. *Manuscript writing*: Hongwei Zeng, Qi Chen, and Ruping Zhao. *Final approval of manuscript*: All authors. Accountable for all aspects of the work: All authors.

## CONFLICT OF INTEREST STATEMENT

The authors declare no conflicts of interest.

## Data Availability

Data will be available on request from the authors.
